# Disease Course After Anti‐CD20 Discontinuation in Secondary Progressive Multiple Sclerosis—A Multicenter Long‐Term Longitudinal Study

**DOI:** 10.1002/acn3.70483

**Published:** 2026-07-28

**Authors:** Ferdinand Otto, Dariia Kliushnikova, Richard Friedrich Radlberger, Sinan Yasaroglu, Tobias Moser, Andrea Harrer, Kitty Kratzer, Wolfgang Hitzl, Christiane Gradl, Martin Schmidauer, Patrick Roth, Harald Hegen, Peter Wipfler

**Affiliations:** ^1^ Department of Neurology Christian‐Doppler University Hospital, Paracelsus Medical University Salzburg Austria; ^2^ Department of Neurology University Hospital Zürich and University of Zurich Zürich Switzerland; ^3^ Department of Dermatology and Allergology University Hospital, Paracelsus Medical University Salzburg Austria; ^4^ Department of Ophthalmology and Optometry Paracelsus Medical University Salzburg Austria; ^5^ Research Program Experimental Ophthalmology & Glaucoma Research Paracelsus Medical University Salzburg Austria; ^6^ Department of Neurology Medical University of St. Pölten St. Pölten Austria; ^7^ Department of Neurology Medical University of Innsbruck Innsbruck Austria

**Keywords:** discontinuation, monoclonal antibodies, ocrelizumab, risk prediction, rituximab, secondary progressive multiple sclerosis

## Abstract

**Objective:**

To describe long‐term outcomes after anti‐CD20 discontinuation in selected patients with secondary progressive multiple sclerosis (SPMS) who remained without subsequent disease‐modifying therapy (DMT).

**Methods:**

We retrospectively analyzed data from four centers in Austria and Switzerland. Inclusion criteria were SPMS, ≥ 2 anti‐CD20 cycles, discontinuation without subsequent DMT, and ≥ 36 months follow‐up. The primary endpoint was time to first confirmed Expanded Disability Status Scale (EDSS) worsening, defined as a ≥ 0.5‐point increase after discontinuation documented at a subsequent routine visit. Secondary endpoints were relapses, MRI activity, and severe infections. No continuation cohort was available.

**Results:**

Fifty‐five patients were included (61% female). Mean age at anti‐CD20 start was 53.5 ± 6.6 years, disease duration 19.3 ± 10.1 years, treatment duration 32.8 ± 16.1 months, and post‐discontinuation follow‐up 55.1 ± 14.1 months. Mean EDSS increased from 5.9 ± 1.3 before anti CD20, to 6.3 ± 1.3 at discontinuation and 6.9 ± 1.2 at last follow‐up. Confirmed EDSS worsening occurred in 31 patients (56%). Hazard analysis suggested an exploratory late increase between 48 and 60 months (*p* = 0.043) based on small numbers at risk. Relapses occurred in 6 patients (10.9%), MRI activity in 4/35 (11%), and severe infections in 17 (31%).

**Conclusions:**

In this selected SPMS cohort, focal inflammatory activity was uncommon, whereas confirmed EDSS worsening accumulated over long‐term follow‐up. Because inclusion required ≥ 36 months untreated follow‐up, early disease activity may have been underestimated, and apparent stability may have been overestimated. Findings are descriptive and hypothesis‐generating and do not establish safety or optimal timing of anti‐CD20 discontinuation.

## 1 Background

Anti‐CD20 monoclonal antibody agents (anti‐CD20) selectively deplete CD20‐expressing B cells [1]. The safety and efficacy of anti‐CD20 in multiple sclerosis (MS) have been demonstrated in several studies and have led to its widespread application in relapsing–remitting MS (RRMS), as well as in the progressive forms, secondary progressive multiple sclerosis (SPMS) and primary progressive MS (PPMS) [2, 3, 4]. Considering that the frequency of MS relapses decreases with age, while therapy‐related adverse events may increase, treatment discontinuation has become an increasingly relevant clinical question [5]. For RRMS patients, there is growing evidence on treatment discontinuation and the risk of new disease activity [6, 7, 8]. However, there is limited evidence regarding cessation of high‐efficacy therapies (HET), particularly anti‐CD20 therapy, in progressive forms of MS. A recent observational study among a heterogeneous RRMS and SPMS cohort older than 50 years with a relatively short average follow‐up of 1.9 years did not find a higher risk of relapses or confirmed disability progression (CDP) in the discontinuation group compared to those who stayed on anti‐CD20 [9]. The duration of the follow‐up is crucial, as B‐cell repopulation may take up to 2 years, suggesting that potential carry‐over effects of anti‐CD20 therapy may persist for a prolonged period after discontinuation [10]. According to guidelines of the European Committee for Treatment and Research in Multiple Sclerosis (ECTRIMS), a short‐term therapeutic trial of anti‐CD20 (so‐called “induction therapy”) may be considered in selected patients with SPMS, although evidence on disease course after discontinuation remains limited [11, 12]. Due to an increasing number of SPMS patients, the question of when to discontinue treatment is becoming increasingly important [13]. Furthermore, although anti‐CD20 therapies are increasingly used in progressive forms of MS, evidence on the clinical course after treatment discontinuation in SPMS remains limited. To address this knowledge gap and to better describe the clinical course after anti‐CD20 discontinuation in patients with SPMS, we conducted a retrospective, multicenter, two‐country follow‐up study. The objective of this study was to investigate the long‐term clinical outcomes after anti‐CD20 discontinuation in a selected cohort of patients with SPMS who remained without subsequent disease‐modifying therapy.

## 2 Methods

### 2.1 Study Design and Participants

This observational, non‐interventional, retrospective, multicenter long‐term study analyzed data from patients at four MS centers in Austria (Innsbruck, St. Pölten, Salzburg) and Switzerland (Zurich) with SPMS treated with anti‐CD20, either rituximab (RTX) or ocrelizumab (OCR), for at least two cycles. At least two cycles were defined as at least two scheduled anti‐CD20 treatment courses administered at the standard 6‐month interval. For OCR, this included the initial split‐dose induction followed by at least one maintenance infusion; for RTX, this included at least two 6‐monthly treatment courses. Anti‐CD20 treatment protocols differed slightly between centers. For rituximab, three of four centers used a regimen of 1000 mg every 6 months (Salzburg, St. Pölten, and Innsbruck), whereas one center used a body surface area‐adjusted regimen of 375 mg/m^2^ (Zürich; also used in selected individual cases in Salzburg). Ocrelizumab was administered according to the approved standard regimen, with two initial doses of 300 mg followed by 600 mg every 6 months. All centers used a standard 6‐month dosing interval and extended‐interval dosing was not applied. The inclusion criteria were age ≥ 45 years at start of anti‐CD20 and diagnosis of SPMS according to the 2014 Lublin criteria at least 1 year prior to initiation of anti‐CD20 [14]. The diagnosis of SPMS was determined by the primary treating neurologist and validated through review of the medical records according to the 2014 Lublin criteria. SPMS required documented progressive disability accumulation independent of relapses, present at least 1 year prior to initiation of anti‐CD20 therapy. Active SPMS was defined as the presence of relapse and/or MRI activity before anti‐CD20 initiation, whereas inactive SPMS was defined as clinical progression without documented relapse or MRI activity before anti‐CD20 initiation. Further inclusion criteria were a clinical follow‐up for at least 36 months after discontinuation of anti‐CD20 without subsequent immunomodulation. Optional data included the availability of MRI scans prior to anti‐CD20, at treatment discontinuation and at follow‐up (> 36 months after the last anti‐CD20 treatment). In addition, B‐cell counts and immunoglobulin levels (prior to anti‐CD20, at discontinuation of anti‐CD20 and at end of follow‐up) were collected where available. Eligible patients had received anti‐CD20 at standard doses, according to the respective treatment protocol of the participating MS centers. Discontinuing treatment was common practice at these centers. At that time, there were no approved treatments for the SPMS course, and this therapy was administered off‐label, typically over a period of 2–3 years. Individual reasons for discontinuation were not systematically recorded across centers, as discontinuation after a limited treatment period reflected common local practice during the study period. The inclusion of patients was approved by the ethics committee of each participating site (Ethics Committee Salzburg, approval number 415‐E/1612/11‐2018, Ethics Committee Zurich BASEC no. 2024‐00239, Ethics committee of the Medical University of Innsbruck, approval number: 1413/2025, Ethics committee Niederösterreich, approval number: GS3‐EK‐4/942‐2025).

### 2.2 Outcomes

The primary endpoint was time to first confirmed EDSS worsening after anti‐CD20 discontinuation. Confirmed EDSS worsening was defined as an increase of ≥ 0.5 EDSS points compared with the EDSS score at anti‐CD20 discontinuation, documented at a subsequent routine follow‐up visit. At the participating MS centers, patients underwent standardized clinical follow‐up including EDSS assessment by MS specialists at least every 6 months. EDSS raters were experienced in the neurological assessment of patients with MS and trained in the use of the EDSS. EDSS worsening was not considered confirmed if it occurred exclusively in the context of an acute relapse, infection, or other transient clinical deterioration. Secondary endpoints included the occurrence of relapses, MRI activity, or severe infections after anti‐CD20 discontinuation. Severe infections were recorded descriptively as part of the post‐discontinuation clinical course and were not used to infer whether anti‐CD20 discontinuation reduced infection risk. Severe infections were defined as infections requiring hospitalization or intravenous antibiotic therapy. For each patient, the participating investigators completed a standardized report form that included information on demographics and clinical characteristics, such as gender, disease duration, previous immunomodulatory treatment, number of relapses, EDSS scores, MRI findings, B‐cell frequencies, immunoglobulin levels, and the occurrence of severe infections. MRI activity was defined as the presence of new T2 lesions or new contrast‐enhancing lesions (CELs). A relapse was defined as a newly developed neurological symptom or reactivation of pre‐existing neurological deficits for at least 24 h in the absence of infection or fever, or as a symptom which occurred at least 30 days after the previous clinical episode [15].

### 2.3 Statistical Analysis

The planned analysis compared the number of relapses, EDSS scores, and changes in EDSS, and MRI activity at treatment initiation, after anti‐CD20, and at the end of follow‐up. Data were checked for consistency and normality using the Shapiro–Wilk test. Fisher's Exact test or Pearson's Chi‐Squared test were used to analyze cross tabulations. In case of normal distributions, independent *t*‐tests were used; otherwise, generalized linear models based on the log‐normal distribution were used to compare means. Cumulative incidences of time to 1st confirmed EDSS worsening were illustrated using Kaplan–Meier curves, and the corresponding hazard rate function was tested whether it remained constant over time or significantly increased using the Gompertz model. All Cox‐regression and Kaplan Meier models were unadjusted for covariates. All reported tests were two‐sided, and *p*‐values < 0.05 were considered statistically significant. All statistical analyses in this report were performed by use of STATISTICA 13 (Cloud Software Group Inc. (2023). Data Science Workbench, version 14.) Data analysis and all statistical analyses were performed by a professional biostatistician (WH).

## 3 Results

### 3.1 Baseline

**FIGURE 1:**
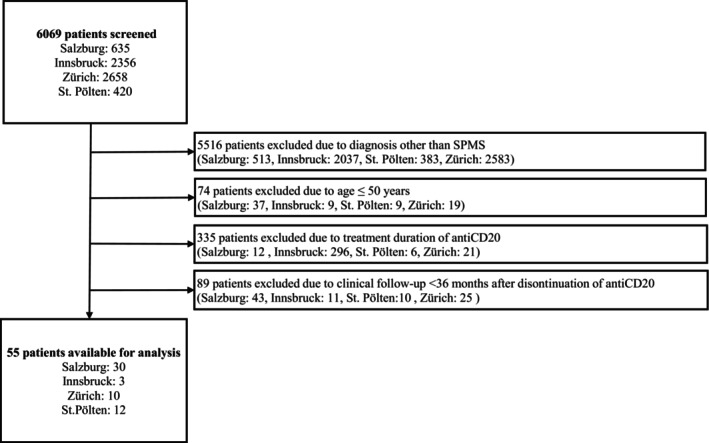
Flow chart of screening and inclusion of patients to the study. anti‐CD20, anti‐CD20 monoclonal antibodies; SPMS, secondary progressive multiple sclerosis.

A total of 6069 patients from four MS centers in Austria and Switzerland were screened for eligibility. Of these, 5516 patients were excluded because of diagnoses other than SPMS, 74 patients were excluded due to age ≤ 45 years, 335 due to anti‐CD20 treatment duration, and 89 because of a clinical follow‐up < 36 months after discontinuation. Ultimately, 55 patients (61% female) were included in the final analysis. Figure 1 illustrates the screening and inclusion process and provides site‐specific counts.

The mean age at inclusion was 53 ± 6.6 years, and the mean disease duration prior to inclusion was 19.3 ± 10.1 years. Patients had received a median of 2 (range 0–5) prior immunomodulatory agents before anti‐CD20 initiation, corresponding to a total of 102 prior disease‐modifying treatment exposures. The most frequently used prior therapies were interferon beta (33/102, 32%), natalizumab (22/102, 22%), fingolimod (17/102, 17%), glatiramer acetate (14/102, 14%), mitoxantrone (9/102, 9%), dimethyl fumarate (4/102, 4%), teriflunomide (2/102, 2%), and cyclophosphamide (1/102, 1%). Thus, prior treatment history included both platform therapies and high‐efficacy or cytotoxic agents, including natalizumab, fingolimod, mitoxantrone, and cyclophosphamide. Anti‐CD20 therapy (45 (82%) received RTX, 10 (18%) OCR) was administered for a mean of 32.8 ± 16.1 months. Reasons for initiating anti‐CD20 included clinical progression (*n* = 46, 84%), relapses (*n* = 13; 24%), and MRI activity (*n* = 28, 51%); multiple reasons could apply to individual patients. In 11 of the 13 patients who experienced a relapse, MRI activity was also observed. Based on relapse and/or MRI activity before anti‐CD20 initiation, 30 patients (54%) were classified as active SPMS and 25 patients (46%) as inactive SPMS. The number of patients receiving RTX (25 in active SPMS vs. 20 inactive SPMS) and OCR (5 vs. 5) was evenly distributed between the subgroups. Baseline and follow‐up characteristics stratified by active versus inactive SPMS are shown in Table S1. No significant differences were observed between the subgroups regarding age, disease duration, number of prior DMTs, follow‐up duration, EDSS at anti‐CD20 initiation, EDSS at discontinuation, or EDSS at the end of follow‐up.

### 3.2 EDSS Progression

The mean follow‐up after anti‐CD20 therapy was 55 months, corresponding to approximately 4.5 years. Mean EDSS prior to anti‐CD20 treatment was 5.9 ± 1.3 (median 6.0, range 3.0–8.0). After anti‐CD20 discontinuation, the mean EDSS was 6.3 ± 1.3 (median 6.5, range: 2.5–8.0) and at the end of follow‐up mean EDSS was 6.9 ± 1.3 (median 7.0, range: 3.0–9.0). The changes in EDSS between the beginning of anti‐CD20 and the end of follow‐up were significant (95% confidence interval: 0.43–0.82, *p* < 0.00001). Confirmed EDSS worsening occurred in 31 patients (56.4%) during the follow‐up period and in 22 patients (40%) during the anti‐CD20 treatment period. Due to the different durations, it was not permissible to compare these two periods. Six patients (10.9%) improved in EDSS during anti‐CD20, whereas none improved after discontinuation. Tables 1 and 2 show baseline and follow‐up characteristics.

**TABLE 1:** Baseline characteristics.

Baseline characteristics prior to initiation of anti‐CD20	
No. of patients	55
Female, no. (%)	34 (62)
Disease duration, years, mean (SD)	19.3 (10.1)
Follow‐up, years, mean (SD)	4.5 (1.2)
Follow‐up, months, mean (SD)	55 (14.1)
Age at anti‐CD20 start, years, mean (SD)	53.5 (6.6)
EDSS (anti‐CD20 therapy—start)	
Mean (SD)	5.9 (1.3)
Median (range)	6.0 (3.0–8.0)
DMTs before anti‐CD20, no. median (range)	2 (0–5)
Anti‐CD20 treatment duration, months, mean (SD)	32.8 (16.1)
cMRI activity (baseline), no. (%)	28 (51)
Relapses (1 year before anti‐CD 20 start), no. (%)	13 (24)

Abbreviations: anti‐CD20, anti‐CD20 monoclonal antibodies; cMRI, cerebral magnetic resonance imaging (MRI activity was defined as the presence of new T2 lesions or new contrast‐enhancing lesions.); DMT, disease‐modifying therapies; EDSS, expanded disability status scale; SD, standard deviation.

**TABLE 2:** Follow‐up characteristics of patients prior, during and after anti‐CD20.

Follow up	
EDSS (start of treatment)	5.9 (1.3)
Mean (SD) median (range)	6.0 (3.0–8.0)
EDSS (end of treatment)	6.3 (1.34)
Mean (SD) median (range)	6.5 (2.5–8.0)
EDSS (anti‐CD20—end of follow‐up)	6.9 (1.23)
Mean (SD) median (range)	7.0 (3.0–9.0)
B cells count/μL (start of treatment) Mean (SD)/%	258.3 (299.2)/13.6
B cells count/μL (end of treatment) Mean (SD)/%	2.0 (8.4)/0.2
B cells count/μL (FU 48 months after treatment) Mean (SD)/%	231.4 (122.7)/14.4
IgG (start of treatment) mean (SD)	977.5 (209.8)
IgG (FU 6 months after end of treatment)	761.4 (233.9)
IgG (FU > 36 months after of treatment)	903.0 (145.5)
IgM (start of treatment) mean (SD)	81.8 (47.1)
IgM (FU 6 months after end of treatment)	55.9 (39.9)
IgM (FU > 36 months after end of treatment)	81.7 (47.2)
IgA (start of treatment) mean (SD)	235.2 (104.8)
IgA (FU 6 months after end of treatment)	182.8 (86.9)
IgA (FU > 36 months after end of treatment)	115.7 (32.2)
cMRI activity (baseline), no. (%)	28 (51)
cMRI activity (during treatment period)	0 (0)
cMRI activity (during FU)	4 (11)
Relapses (baseline), no. (%)	13 (24)
Relapses (during treatment period)	0 (0)
Relapses (during FU)	6 (11)
Severe infection during FU, no. of patients (%)	17 (31)

Abbreviations: anti‐CD20, anti‐CD20 monoclonal antibodies; cMRI, cerebral magnetic resonance imaging; DMT, disease‐modifying therapies; EDSS, expanded disability status scale; FU, follow‐up; Ig, immunoglobulin; SD, standard deviation.

**FIGURE 2:**
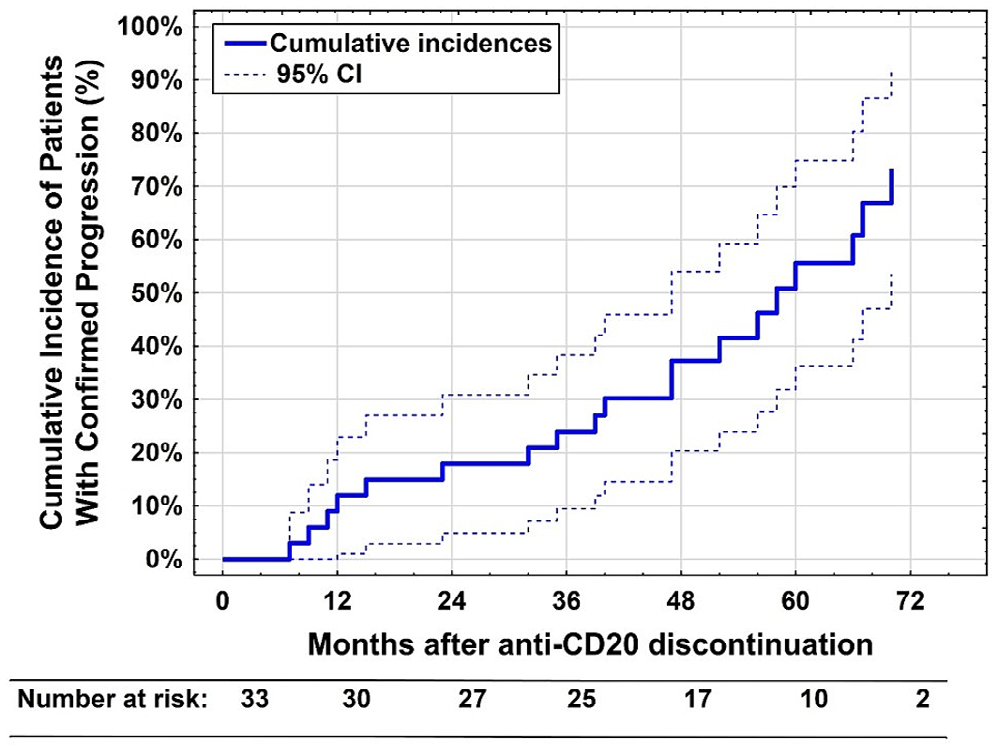
Cumulative incidence of first confirmed EDSS worsening within 72 months follow‐up after anti‐CD20 discontinuation.

**FIGURE 3:**
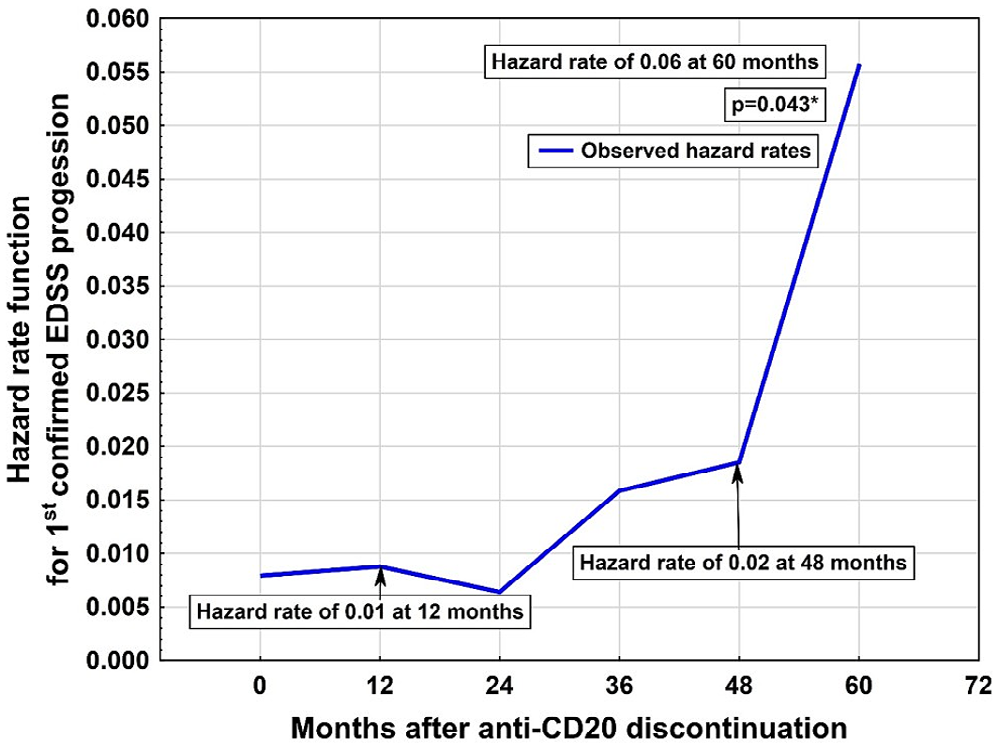
Hazard rates for first confirmed EDSS worsening after anti‐CD20 discontinuation at 0, 12, 24, 36, 48, and 60 months with statistically increasing hazard rates over time (*p* = 0.043).

To analyze the time to first confirmed EDSS worsening after anti‐CD20 discontinuation, we plotted the cumulative incidences over time (Figure 2). Corresponding hazard rates were also plotted (Figure 3), and statistical tests revealed that hazard rates significantly increased over time (*p* = 0.043), indicating increased risk for confirmed progression over time. A detailed analysis demonstrated that 12 months after discontinuation of anti‐CD20, the corresponding hazard rate for progression in terms of EDSS worsening was low (0.01) and remained low until 48 months after discontinuation of anti‐CD20 with 0.02. Hazard rate at 60 months after discontinuation increased up to 0.06, demonstrating an increased risk for EDSS progression between 48 and 60 months after anti‐CD20 discontinuation (Figure 2).

Figure 3 shows the hazard rates for first confirmed EDSS worsening at a given time. Since the hazard rate at 48 months after discontinuation rose to 0.06, it is suggestive that there is an increased risk for EDSS progression between 48 and 60 months after discontinuation of anti‐CD20. The hazard rates for first confirmed EDSS worsening were found to significantly differ from a constant function over time (*p* = 0.043).

To determine whether confirmed EDSS worsening differed between active and inactive SPMS patients after anti‐CD20 discontinuation, we conducted a subgroup analysis. There was no significant difference in confirmed EDSS worsening over time between the active and inactive subgroups (see Figure S1).

### 3.3 Relapses and MRI Activity

In 13 (24%) patients experienced a relapse before the start of anti‐CD20; during treatment only one relapse was documented in one patient. During the follow‐up period, in six patients (10.9%) occurred a relapse, including one patient with two relapses. Prior to inclusion, 28 patients (51%) showed MRI activity (defined as a new T2 lesions or new CEL). Among those with available MRI follow‐up, no patient exhibited new T2 or CEL at the time of anti‐CD20 discontinuation. MRI data during treatment was not available for 15 patients (27.3%). At the end of follow‐up, MRI scans were performed in 35 patients of whom 31 (89%) patients remained radiologically stable, while four patients (11%) showed new T2 or CEL.

### 3.4 B‐Cell Levels, Immunoglobulins, Infections

The mean B‐cell count (*n* = 33) prior to anti‐CD20 initiation was 258/μL ± 299 cells/μL (normal range 60–800 cells/μL), which dropped to 2 ± 8 cells/μL during treatment. At follow‐up (48 months after the last anti‐CD20 treatment), the B‐cell count had returned to near‐baseline levels (231 ± 123 cells/μL) (see Table 2).

In an exploratory analysis, we assessed whether the timing of peripheral total B‐cell repopulation was associated with confirmed EDSS worsening after anti‐CD20 discontinuation. B‐cell repopulation was operationally defined as recovery of total peripheral B cells to at least 50% of the individual pre‐treatment baseline value. This analysis did not show a statistically significant association between earlier B‐cell repopulation and confirmed EDSS worsening (*p* = 0.22; Figure S2). Memory B‐cell subsets were not systematically available and could therefore not be analyzed.

IgG hypogammaglobulinemia was observed in 36% of the patients (defined as IgG < 600 mg/dL 6 months after the last anti‐CD20 infusion). After the follow‐up period of more than 36 months, mean IgG levels (903 ± 146 mg/dL) returned to near‐baseline values (978 ± 210 mg/dL), following a temporary decrease 6 months post‐treatment (761 ± 234 mg/dL; *p* < 0.01). A significant temporary decrease was also observed for IgM, with recovery to baseline values at the end of follow‐up (see Table 2). In contrast, IgA levels did not fully recover and remained significantly reduced at the end of the follow‐up (Table 2). Severe infections were noted in 17 (31%) patients during follow‐up. No clear association was found between B‐cell counts or hypogammaglobulinemia and severe infections. However, this analysis was limited by the small number of available B‐cell and immunoglobulin measurements in patients with infections and by the absence of multivariable adjustment.

## 4 Discussion

In this multicenter, two‐country retrospective cohort study, we investigated long‐term clinical outcomes of 55 patients with SPMS after discontinuation of anti‐CD20 therapy. Over a mean follow‐up of 55 months (4.5 years) after treatment cessation, focal inflammatory disease activity remained low: only 6 of 55 patients (10.9%) experienced relapses, and MRI findings were stable in 31 of 35 patients (89%). However, confirmed EDSS worsening accumulated during long‐term follow‐up, and the non‐constant hazard analysis showed an exploratory increase beyond 48 months after discontinuation. Given the absence of a contemporaneous continuation cohort or natural history comparator, this observation cannot be attributed to anti‐CD20 discontinuation or to waning anti‐CD20 carry‐over efficacy. It may also reflect the natural course of SPMS, aging, high baseline disability, prior treatment exposure, comorbidities, or other unmeasured factors. Our findings should therefore not be interpreted as evidence for or against continuation of anti‐CD20 therapy in patients with SPMS.

Since data on HET discontinuation in SPMS are limited, only limited conclusions can be drawn from heterogeneous study populations including both RRMS and SPMS patients. Our results are in line with a recent observational cohort study that analyzed discontinuation of HET, including anti‐CD20 agents, among patients aged > 50 years with RRMS (*n* = 100) and SPMS (*n* = 68) who had shown no relapse or MRI activity for at least 2 years during treatment, compared with a control group continuing HET [9]. The study found that anti‐CD20 therapy maintained its beneficial effect on relapse reduction and disability stabilization for at least 18 months after discontinuation. However, from year 3 onward, there was a trend toward increased progression in the discontinuation group compared with those who stayed on the therapy. In contrast to our findings, the mean follow‐up in that study was shorter (1.9 years), which may explain why a later increase in progression was not detected. Another multicenter retrospective study of a heterogenous cohort (*n* = 53)—mostly RRMS with only 12 progressive MS patients—reported low disease activity in 9.8% (*n* = 4) after discontinuation [16]. However, the mean follow‐up of only 16 months limits the interpretation of long‐term effects on progression. Similar findings were reported by Konen et al., who showed sustained over 30 months in patients with RRMS after ocrelizumab discontinuation [8]. In contrast to the cited literature, our study focused exclusively on patients with SPMS and included substantially longer follow‐up, enabling detection of the late increase in disability progression. However, because no continuation cohort or natural history comparator was included, this observation cannot be causally attributed to treatment discontinuation and should be regarded as hypothesis‐generating. One of the few studies focusing exclusively on patients with SPMS is a retrospective multicenter analysis of RTX treatment, with a sample size (*n* = 54) comparable to ours. That study found a significantly delayed confirmed progression compared with matched RTX‐naïve SPMS controls, and lower EDSS scores after up to 10 years of follow‐up [17]. Consistent with our findings, the authors concluded that B‐cell depletion with anti‐CD20 represents a therapeutic option for patients with SPMS showing inflammatory activity, leading to slower progression. However, that study did not address whether or when patients who discontinue anti‐CD20 treatment return to the pre‐treatment rate of progression.

The exploratory analysis of peripheral total B‐cell repopulation did not show a significant association with confirmed EDSS worsening after anti‐CD20 discontinuation. This finding should be interpreted cautiously. Recovery to 50% of the individual pre‐treatment B‐cell count was used as an operational marker of peripheral B‐cell reconstitution based on available routine clinical data, but it does not represent a validated biological threshold for disability worsening in SPMS. Moreover, only total peripheral B‐cell counts were available, whereas memory B‐cell subsets, which may be more relevant for inflammatory disease activity after anti‐CD20 therapy, were not systematically assessed. Therefore, our data do not allow conclusions regarding the relationship between B‐cell subset reconstitution and disability accumulation after anti‐CD20 discontinuation. We also observed a significant reduction in all three immunoglobulin classes (IgG, IgM, IgA) following anti‐CD20, a return to baseline levels of IgG and IgM after discontinuation, and a persistent decrease for IgA. Whether this persistent IgA decrease contributes to disease progression or infections remains speculative. A recent review suggests that IgA may play a role in MS disease progression [18].

A notably high proportion of severe infections (31%, *n* = 17) was observed during follow‐up. This finding is clinically relevant but should be interpreted as descriptive safety observations only. Because no continuation cohort was available and no formal pre−/post‐discontinuation person‐time analysis was performed, our study cannot determine whether infection risk decreased after stopping anti‐CD20 therapy or whether it differed from patients who continued treatment. Similarly, although lymphopenia and hypogammaglobulinemia have previously been linked to infection risk [19], our cohort does not allow firm conclusions regarding the relationship between immune reconstitution, immunoglobulin recovery, and severe infections. Interpretation is limited by the retrospective design, the small number of infection events, incomplete availability of immunoglobulin and B‐cell measurements at the time of infection, and the absence of adjustment for age, disability level, comorbidities, steroid exposure, prior treatment exposure, and cumulative anti‐CD20 dose. Accordingly, infection outcomes should be regarded as descriptive and hypothesis‐generating. Although constrained by the small cohort size, our findings are broadly consistent with larger studies identifying infection risk as a relevant concern during and after anti‐CD20 therapy [20].

Among the study's strengths are the long follow‐up period, which makes it possible to detect a potential late increase in the risk of disease progression (> 48 months), the multicenter study design conducted under real‐world conditions, and the exclusive focus on SPMS patients in an area where there is currently limited evidence. However, our study also has several important limitations. First, the retrospective observational design precludes causal inference. Although predefined inclusion criteria and harmonized definitions across centers were applied, residual confounding cannot be excluded. The lack of a continuation cohort is a central limitation of this study. Without a comparator group, we cannot determine whether EDSS worsening after discontinuation reflects treatment cessation, the expected natural course of SPMS, high baseline disability, or other unmeasured confounders. Second, the inclusion criterion of at least 36 months of follow‐up after discontinuation may have introduced immortal time or survivorship bias. Patients had to remain under observation long enough to be included, which may have selected for individuals with sufficient clinical stability, survival, or continued follow‐up within participating centers. This may have led to an underestimation of early adverse outcomes after discontinuation and limits the generalizability of our findings to all patients stopping anti‐CD20 therapy. Third, a further consequence of conditioning inclusion on at least 36 months of untreated follow‐up is that early post‐discontinuation disease activity may have been underestimated, whereas the apparent stability of remaining untreated may have been overestimated. This limitation is particularly relevant for interpreting the relapse and MRI activity rates observed in our cohort. However, in the specific clinical context of this study, we consider the potential impact of this bias to be limited. During the study period, no approved alternative disease‐modifying therapy was available for patients with established, predominantly progressive SPMS and high disability levels. Therefore, although early disease activity or treatment restart may have been missed in individual cases, systematic exclusion of a large group of patients who rapidly switched to another approved therapy appears unlikely. Nevertheless, our findings should be interpreted as applying only to selected patients with SPMS who remained untreated after anti‐CD20 discontinuation and not to all patients stopping anti‐CD20 therapy. Fourth, confounding by indication is possible. Reasons for discontinuation were not systematically defined and may have included clinical stability, advanced age, disability progression despite treatment, infections, hypogammaglobulinemia, patient preference, or physician concerns regarding long‐term immunosuppression. These factors are themselves associated with subsequent disability progression and infection risk and may therefore have influenced outcomes independently of treatment discontinuation. Because we did not apply a multivariable statistical model or propensity‐score approach to adjust for these baseline differences, our results should be interpreted as descriptive rather than causal.

In addition, the observed increase in the hazard of confirmed EDSS worsening beyond 48 months was based on a limited number of patients remaining at risk at later time points. Therefore, this finding should be considered exploratory and requires confirmation in larger prospective cohorts with longer and more complete follow‐up. Although EDSS assessments were performed routinely by trained MS specialists at the participating centers, the use of a ≥ 0.5‐point EDSS increase as the primary disability endpoint remains a limitation. Small EDSS changes may be vulnerable to inter‐rater variability, particularly in a retrospective multicenter study and in patients with high baseline disability. We attempted to reduce this limitation by considering EDSS worsening only when documented during routine follow‐up and not attributable to relapse, infection, or other transient clinical deterioration. Nevertheless, the absence of a formal prospective adjudication procedure and the lack of a stricter sensitivity analysis using a higher EDSS threshold limit the interpretation of disability progression. Regarding imaging, 15 patients lacked MRI follow‐up during treatment and 20 had no MRI at final follow‐up, which may have led to an underestimation of subclinical inflammatory activity. Retrospective data from SPMS cohorts with similar demographics have shown MRI stability in approximately 65% of patients, while radiological disease activity persisted in 35%; among those receiving anti‐CD20 therapy, only 2 of 5 displayed new MRI activity, consistent with our findings [21]. Furthermore, anti‐CD20 treatment protocols were not fully uniform across centers. Although all centers used standard 6‐month dosing intervals, rituximab dosing differed between fixed‐dose regimens and body surface area‐adjusted dosing in some centers, whereas ocrelizumab was administered according to the approved standard regimen. We therefore cannot exclude center‐specific treatment protocols, cumulative anti‐CD20 exposure, or differences between rituximab and ocrelizumab that may have influenced treatment effects, immune reconstitution, infection risk, or post‐discontinuation outcomes. The only currently available meta‐analysis of RTX in SPMS does not differentiate between different treatment protocols, which may contribute to heterogeneity across studies [22].

Our findings highlight the need for prospective, controlled studies to determine the optimal timing of anti‐CD20 discontinuation in SPMS, and to better understand the biological mechanisms underpinning prolonged carry‐over and subsequent rise in disability risk. Future studies should include biomarker‐guided disease activity monitoring, such as serum neurofilament light chain (Nfl) and serum glial fibrillary acidic protein (GFAP) [23, 24]. Moreover, future trials should use a composite endpoint combining EDSS, the 9‐Hole Peg Test, and the Timed 25‐Foot Walk Test to ensure that treatment effects on hand function and walking function are not overlooked—as emphasized in recent recommendations and the O'HAND study evaluating OCR in primary progressive MS [25, 26].

To conclude, this retrospective multicenter study describes long‐term outcomes in a selected cohort of patients with SPMS who discontinued anti‐CD20 therapy and remained without subsequent DMT. Within this selected population, relapses and MRI activity were uncommon, whereas confirmed EDSS worsening accumulated during long‐term follow‐up. Because inclusion was conditioned on at least 36 months of untreated follow‐up, early post‐discontinuation disease activity may have been underestimated and the apparent stability of continued non‐treatment may have been overestimated. These findings remain descriptive and hypothesis‐generating and should not be interpreted as proof of the safety or optimal timing of anti‐CD20 discontinuation.

## Author Contributions

**Ferdinand Otto:** formal analysis, investigation, methodology, project administration, visualization, writing – original draft, writing – review and editing. **Dariia Kliushnikova:** data curation, formal analysis, investigation, project administration, writing – review and editing. **Richard Friedrich Radlberger:** formal analysis, methodology, validation, writing – review and editing. **Sinan Yasaroglu:** formal analysis, methodology, validation, writing – review and editing. **Tobias Moser:** formal analysis, methodology, validation, writing – review and editing. **Andrea Harrer:** conceptualization, formal analysis, validation, writing – review and editing. **Kitty Kratzer:** formal analysis, validation, writing – review and editing. **Wolfgang Hitzl:** data curation, formal analysis, investigation, methodology, validation, visualization, writing – review and editing. **Christiane Gradl:** conceptualization, formal analysis, validation, writing – review and editing. **Martin Schmidauer:** conceptualization, formal analysis, validation, writing – review and editing. **Patrick Roth:** conceptualization, formal analysis, validation, writing – review and editing. **Harald Hegen:** conceptualization, formal analysis, validation, writing – review and editing. **Peter Wipfler:** conceptualization, data curation, formal analysis, investigation, methodology, project administration, supervision, validation, writing – original draft, writing – review and editing.

## Funding

The authors have nothing to report.

## Conflicts of Interest

The authors declare no conflicts of interest.

## Supporting information


**Figure S1:** Cumulative incidence of first confirmed EDSS worsening in active versus inactive SPMS patients within 72 months after anti‐CD20 discontinuation.
**Figure S2:** Kaplan–Meier curve for the cumulative proportions of first EDSS progression with the time to repopulation of B cells to 50% of the individual pre‐anti‐CD20 treatment levels.
**Table S1:** Baseline and follow‐up characteristics stratified by active versus inactive SPMS before anti‐CD20 initiation. Active SPMS was defined as relapse and/or MRI activity before anti‐CD20 initiation, whereas inactive SPMS was defined as clinical progression without documented relapse or MRI activity before anti‐CD20 initiation. Data are presented as mean (standard deviation) median (range), or number (%), as appropriate. anti‐CD20, anti‐CD20 monoclonal antibody therapy; cMRI, cerebral magnetic resonance imaging; DMT, disease‐modifying therapy; EDSS, expanded disability status scale; FU, follow‐up; mo, months; ns, not significant; OCR, ocrelizumab; RTX, rituximab; SD, standard deviation; SPMS, secondary progressive multiple sclerosis.

## Data Availability

The data that support the findings of this study are available on request from the corresponding author. The data are not publicly available due to privacy or ethical restrictions.
