# Supplementary material for: Disease Course After Anti‐CD20 Discontinuation in Secondary Progressive Multiple Sclerosis—A Multicenter Long‐Term Longitudinal Study
Source: Ann Clin Transl Neurol. 2026 Jul 28:10.1002/acn3.70483. Online ahead of print. doi: 10.1002/acn3.70483 (PMC13416117; doi:10.1002/acn3.70483)
Supplement: Supplementary file 1 — Figure S1: Cumulative incidence of first confirmed EDSS worsening in active versus inactive SPMS patients within 72 months after anti‐CD20 discontinuation. Figure S2: Kaplan–Meier curve for the cumulative proportions of first EDSS progression with the time to repopulation of B cells to 50% of the individual pre‐anti‐CD20 treatment levels. Table S1: Baseline and follow‐up characteristics stratified by active versus inactive SPMS before anti‐CD20 initiation. Active SPMS was defined as relapse and/or MRI activity before anti‐CD20 initiation, whereas inactive SPMS was defined as clinical progression without documented relapse or MRI activity before anti‐CD20 initiation. Data are presented as mean (standard deviation) median (range), or number (%), as appropriate. anti‐CD20, anti‐CD20 monoclonal antibody therapy; cMRI, cerebral magnetic resonance imaging; DMT, disease‐modifying therapy; EDSS, expanded disability status scale; FU, follow‐up; mo, months; ns, not significant; OCR, ocrelizumab; RTX, rituximab; SD, standard deviation; SPMS, secondary progressive multiple sclerosis. [file ACN3-9999-0-s001.docx]

**Supplementary Material for Review**


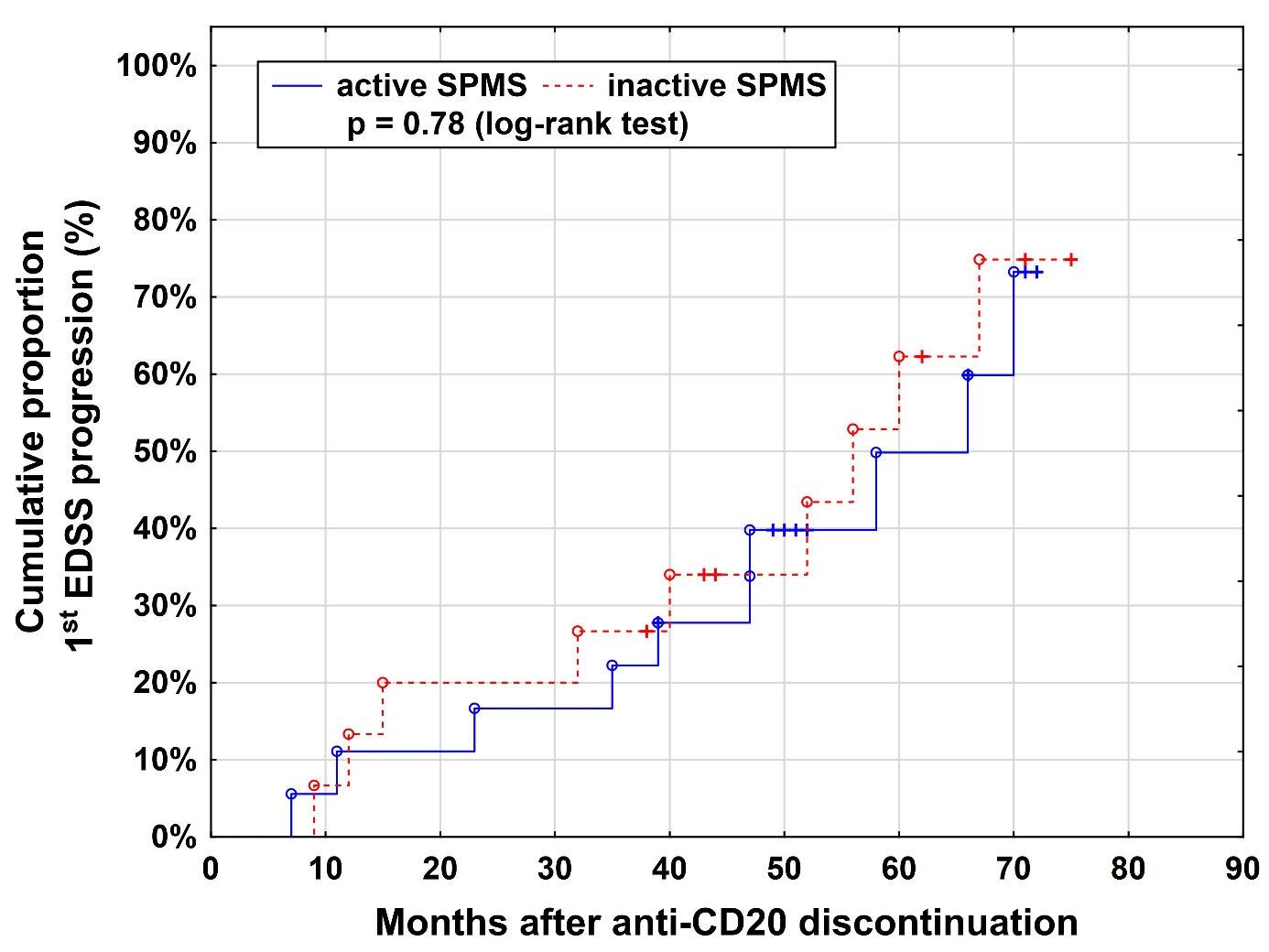


**Supplementary Figure 1** Cumulative incidence of first confirmed EDSS worsening in active versus inactive SPMS patients within 72 months after anti-CD20 discontinuation.


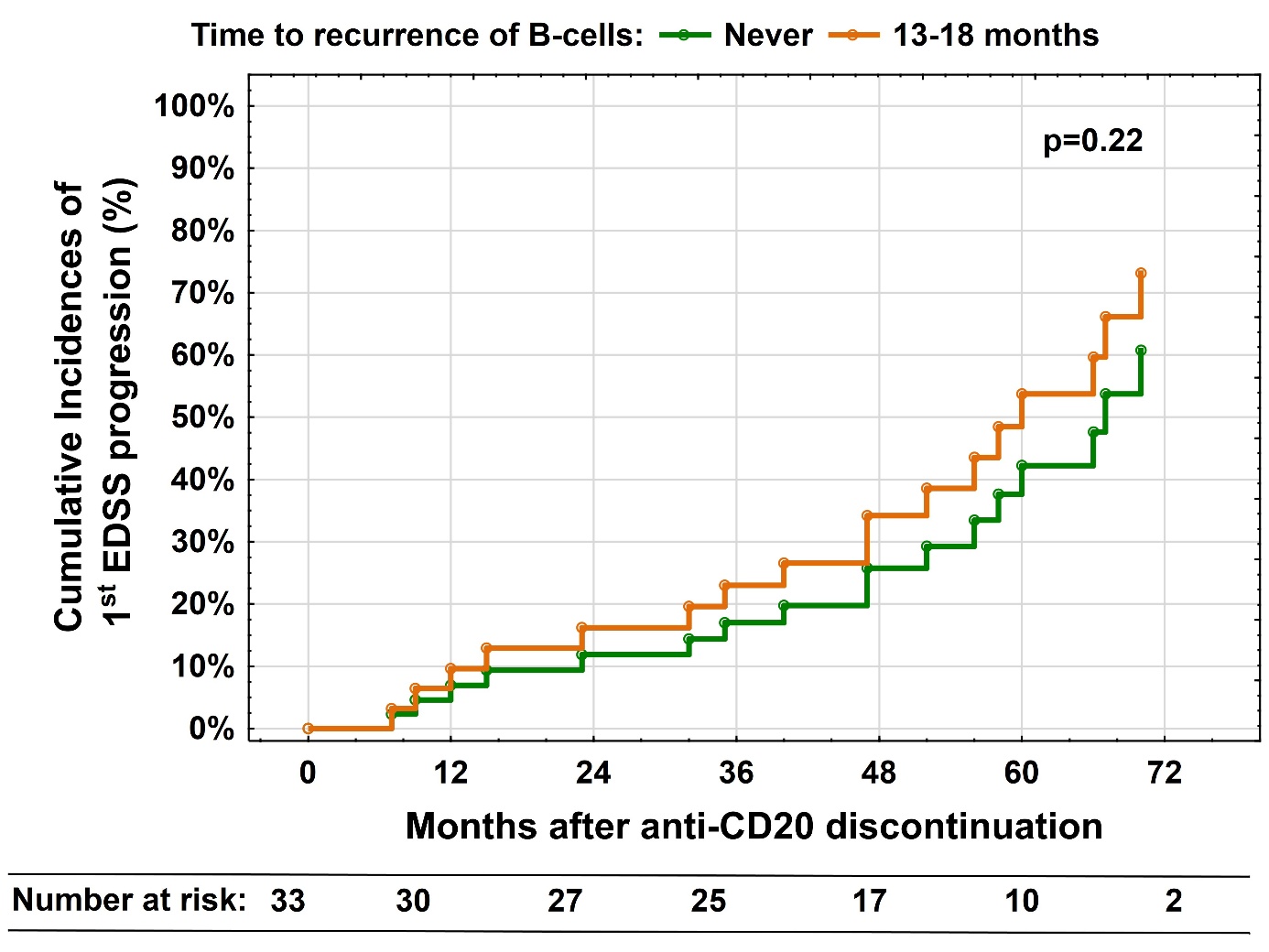
**Supplementary Figure 2** Kaplan-Meier curve for the cumulative proportions of first EDSS progression with the time to repopulation of B cells to 50% of the individual pre-anti-CD20 treatment levels

| Baseline characteristics |  | active SPMS | Inactive SPMS |  |
| --- | --- | --- | --- | --- |
| No. of patients |  | 30 | 25 | ns |
| Female, No (%) |  | 18 (60) | 16 (64) | ns |
| Disease duration, y Mean (SD) |  | 19.4 (9) | 19.2 (11) | ns |
| Follow-up, mo  Mean (SD) |  | 56 (14.4) | 53 (13.3) | ns |
| DMTs before antiCD20, No  Median (range) |  | 2 (0-4) | 2 (0-5) | ns |
| Age at antiCD20 start, y  Mean (SD) |  | 52.5 (6.2) | 54.9 (6.7) | ns |
| EDSS (start of antiCD20)  Mean (SD)  Median (range) |  | 5.91 (1.3)  6.0 (3.0-8.0) | 5.90 (1.1)  6.0 (3.5-8.0) | ns |
| EDSS (end of antiCD20)  Mean (SD)  Median (range) |  | 6.28 (1.3)  6.5 (3.5-8.0) | 6.22 (1.3)  6.5 (2.5-8.0) | ns |
| EDSS (end of FU)  Mean (SD)  Median (range) |  | 6.96 (1.1)  7.0 (3.5-8.5) | 6.78 (1.3)  7.0 (3.0-9.0) | ns |
| MRI activity (baseline), No (%) |  | 28 (93) | 0 |  |
| Relapses (1 y before antiCD20 start), No (%) |  | 13 (43) | 0 |  |

**Supplementary Table 1**: Baseline and follow-up characteristics stratified by active versus inactive SPMS before anti-CD20 initiation. Active SPMS was defined as relapse and/or MRI activity before anti-CD20 initiation, whereas inactive SPMS was defined as clinical progression without documented relapse or MRI activity before anti-CD20 initiation. Data are presented as mean (standard deviation) median (range), or number (%), as appropriate. SPMS: secondary progressive multiple sclerosis; EDSS: Expanded Disability Status Scale; anti-CD20: anti-CD20 monoclonal antibody therapy; RTX: rituximab; OCR: ocrelizumab; DMT: disease-modifying therapy; cMRI: cerebral magnetic resonance imaging; FU: follow-up; mo: months; ns: not significant; SD: standard deviation.
